# Netherton Syndrome: A Systematic Review of the Challenges of Diagnosis and Treatment

**DOI:** 10.7759/cureus.98322

**Published:** 2025-12-02

**Authors:** Maximus S Reese, Ryan Nguyen, Noor Chughtai, Philip J Haynos, Salma Alkhatib, Shantanu Amin, Ethan Speer, Jared Nichols

**Affiliations:** 1 College of Medicine, Kansas City University, Joplin, USA; 2 College of Medicine, Orlando College of Osteopathic Medicine, Winter Garden, USA; 3 Osteopathic Manipulative Medicine, Kansas City University, Joplin, USA

**Keywords:** biallelic mutations, genodermatosis, immune dysregulation syndrome, lekt1, spink5

## Abstract

Netherton syndrome is an autosomal recessive genodermatosis caused by biallelic mutations in the SPINK5 gene, a gene that codes for lymphoepithelial Kazal-type-related inhibitor 1 (LEKT1) protein. This disorder is characterized by erythroderma, ichthyosis, hair shaft abnormalities, and immune dysregulation. While there is increasing research and recognition of Netherton syndrome, much remains unknown, with limited knowledge of the history, manifestations, and responses to therapies. The purpose of this study was to review case reports of Netherton’s to draw conclusions from patient demographics, symptoms, treatment, and outcomes. A systematic review of 30 case reports and clinical studies on Netherton syndrome was conducted, including only patient-level clinical data of studies in English. Data were extracted via full-text review, organized into comparative tables, and analyzed for common patterns in symptoms, diagnostic techniques, and treatments. Biologic therapies consistently reduced Netherton syndrome effects with improvements in symptoms and infection rates. No cases of complete remission have been reported from the cases examined. With no standardized treatment in place, larger studies are needed. While biologic therapies have offered improvement of symptoms for patients with Netherton syndrome, remission is still not achievable. Treatment outcomes are variable and require an individualized technique in patient care. Genetic testing is the most valuable for diagnosis, but it is not always accessible. Lack of standardized therapy exhibits a need for larger studies to be conducted in order to improve patient care.

## Introduction and background

**Figure 1:**
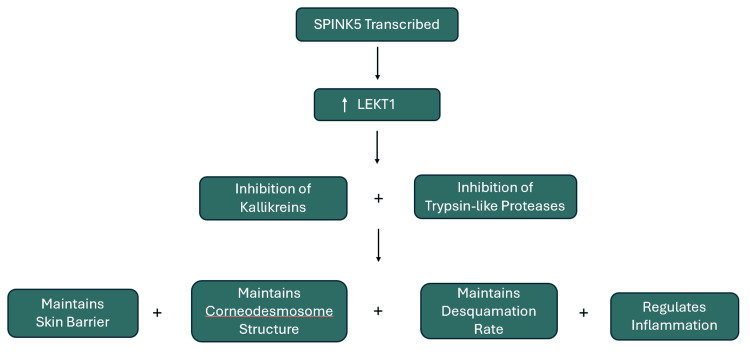
SPINK5 gene pathway. LEKT1: lymphoepithelial Kazal-type-related inhibitor 1.

Netherton syndrome (NS) is a rare, autosomal recessive genodermatosis disorder caused by biallelic mutations in the SPINK5 gene. This gene encodes the protein lymphoepithelial Kazal-type-related inhibitor 1 (LEKT1), a serine peptidase inhibitor that controls the activity of certain serine peptidase enzymes responsible for the breakdown of other proteins in the outer layer of the skin (stratum corneum) [1]. LEKT1 also plays a role in normal hair follicle development, the development of lymphocytes, and the control of peptidases that trigger immune system function. Mutations in SPINK5 result in decreased LEKT1 allowing for increased breakdown of proteins in the epidermis resulting in excessive skin shedding, abnormal hair growth, and immune dysfunction [1,2]. A summary of the SPINK5 gene's role can be seen in Figure 1. 

Netherton's is classically characterized by erythroderma (diffuse, red skin at birth or early infancy), ichthyosis (scaly skin), hair shaft abnormalities (trichorrhexis invaginata, “bamboo hair”), and immune dysregulation (elevated IgE, eosinophilia, recurrent infections). NS can become severe due to life-threatening electrolyte imbalances, dehydration, or because of the recurrent infections, which exhibit that this disease can have a significant impact on quality of life [3-5].

**Figure 2:**
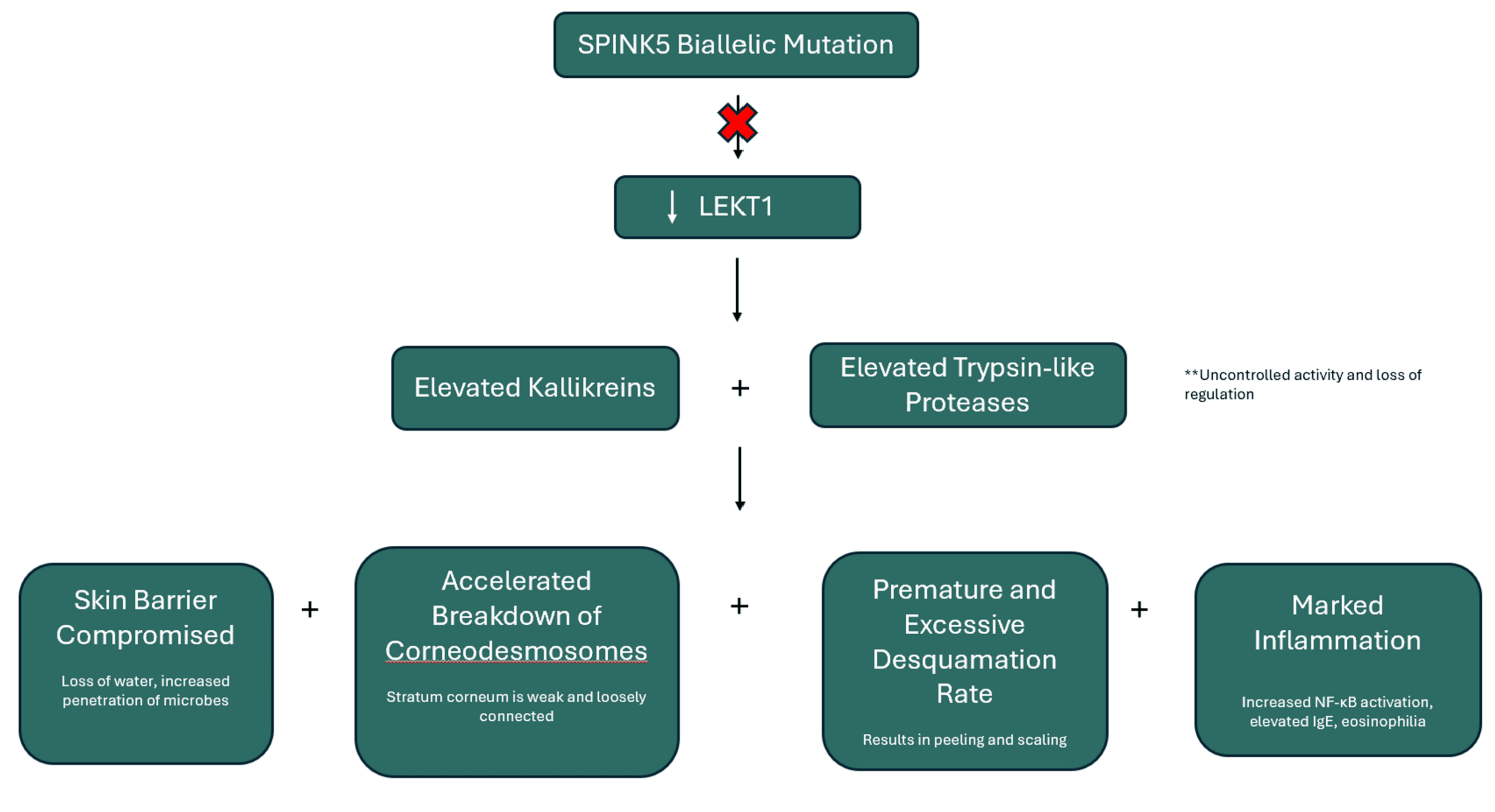
SPINK5 gene mutation pathway. LEKT1: lymphoepithelial Kazal-type-related inhibitor 1.

Being a rare disease, much remains unknown about why NS manifests, what therapies best work for it, and how to best distinguish it from other severe dermatological disorders. Pathophysiology is poorly understood, and its clinical phenotype is variable. Many symptoms overlap with other severe dermatological disorders, which complicates diagnosis. While there are no current standardized therapies, accurate identification can rule out inappropriate treatment options and help patients receive care that may be beneficial sooner. Molecular genetic testing for the SPINK5 mutation is known to be key in confirming NS, but resources are variable, and this test cannot always be ordered. Presently, much of the treatment for NS is supportive, but emerging targeted therapies have potential in individualized and successful care. Further study and research is needed to better understand disease mechanisms and develop evidence-based treatment strategies [6]. A depiction of the SPINK5 gene mutation pathway is seen in Figure 2.

## Review

Methods 

A systematic review of existing case reports and clinical studies involving patients with Netherton syndrome was performed with a focus on patient-level data. A total of 34 patients from 30 studies were included, with articles being retrieved from the PubMed database. The search term "Netherton syndrome" was used with filters limiting results to published case reports within the past 10 years. This search retrieved 52 articles, of which 30 were ultimately selected based on their relevance and variability in treatment approaches to allow for cross-analysis. Additional reports of Netherton syndrome were identified but excluded due to incomplete clinical data. Relevant case reports may exist that were not included; therefore, the findings of this review should be considered for the sample collected. 

Inclusion criteria for this review required studies with patient-level clinical data on subjects, including the patient’s medical history, presenting symptoms, previous treatments, diagnostic methods, treatments administered, and patient outcomes. Exclusion criteria included non-English reports without translation and papers lacking sufficient clinical detail of patient history, presentation, or diagnosis. To extract data for this report, full-text reviews were performed for each article considered. Data were organized into comparative tables and categories based on presentation, diagnostic techniques, lab findings, and treatment. Findings for the review were synthesized by identifying common patterns across reports, diagnostic challenges highlighted, exhibited treatment effectiveness, and important physician insights noted in managing this disease. All included studies were case reports; however, no formal risk-of-bias or quality assessment was performed. Potential bias and limitations of this review are considered in the interpretation of the findings.

Results

Thirty-four patients were identified across 30 case reports, including those from studies reporting multiple affected individuals. Patient ages ranged from approximately one month to 62 years, the age gap reflecting when patients were looking for definitive treatment. Gender distribution was as follows: 23 males (67.65%) and 11 females (32.35%). Two adult Bahraini siblings (58 year old female and 62 year old male), a 27 month old Omani male, and a seven year old Algerian female were reported in the data to be born to consanguineous parents -- these cases highlighted the autosomal recessive inheritance pattern of Netherton Syndrome (11.76% of cases presented from first degree consanguineous parents) [7-9]. Patients were globally distributed, representing the Middle East, Asia, Europe, and North America. Tables 1-4 summarize the analysis of reviewed cases, including presentations, lab findings, treatments, and key findings (see Appendix, Table 5).

**Table 1:** Clinical symptoms in Netherton syndrome cases (N=34). *Seven cases did not identify or disprove the finding of trichorrhexis invaginata.

Clinical symptom	Number of cases presenting
Erythroderma/reddish skin/generalized erythema	34
Hair abnormalities (general: thin, fragile, sparse, brittle, lusterless, short)- “Bamboo Hair”-trichorrhexis invaginata	27*
Scaling/desquamation	16
Recurrent infections	15
Pruritus	18
Atopic manifestations (allergies, asthma)	17
Ichthyosis linearis circumflexa (ILC)	10

**Table 2:** Summary of diagnostic approaches and tools used in reviewed studies. WES: whole exome sequencing; LEKTI: lymphoepithelial Kazal-type-related inhibitor; PRP: pityriasis rubra pilaris.

Diagnostic category	Common methods used
Genetic testing	SPINK5 sequencing, WES, qRT-PCR, Sanger sequencing
Hair analysis	Light/electron microscopy, trichoscopy
Skin biopsy	Psoriasiform hyperplasia, parakeratosis, spongiosis
Immunohistochemistry	LEKTI, IL-36, cytokine markers
Lab tests	IgE ↑, eosinophils ↑, vitamin D ↓, WBC ↑
Imaging	Chest X-ray, bone age, echo, brain MRI
Immune/allergy testing	Flow cytometry, IgE panels
Misdiagnosis cases	Atopic dermatitis, ichthyosis, PRP

**Table 3:** Diagnostic findings in Netherton syndrome cases (N=34). LEKTI: lymphoepithelial Kazal-type-related inhibitor.

Diagnostic finding	Number of cases presenting
Elevated IgE	28
Genetic testing confirming SPINK5 mutation	26
Immunohistochemistry staining for LEKTI	6
Hyperplasia	6
Hyperkeratosis	10
Spongiosis	13

**Table 4:** Treatments used in Netherton syndrome cases. *Surgery, IV fluids, oral contraceptives, and zinc oxide paste were noted to be administered in cases, but for conditions unrelated to Netherton syndrome.

Treatment type	Number of cases using treatment	Cases with reduced symptoms	Cases with failed treatment	Adverse effects
Immunosuppressants (topical corticosteroids)	22	8	14	Immune system suppression; increased infection risk
Antihistamines	9	5	4	Sedation
Antibiotics (systemic or topical)	8	5	3	GI upset; vulnerable to certain infections
Dupilumab	8	5	3	No adverse effects noted in adult or infant
Omalizumab	1	1	0	No adverse effects noted
Infliximab	1	1	0	No adverse effects noted
Pembrolizumab	1	1	0	In previous studies, pembrolizumab showed various adverse effects involving multiple organ systems. In this report, no adverse events were noted
Secukinumab*	6	4	2	A potential adverse effect from treatment was noted in one of the cases; however, it was later found negligible due to the same findings in the absence of treatment. The other cases of secukinumab treatment involved no adverse effects
Nutritional support/ supplements/allergen-free diet	8	5	3	No adverse effects noted
Acitretin (oral retinoid)	5	2	3	No adverse effects noted
Abrocitinib	1	1	0	No adverse effects noted
Tofacitinib	1	0	0	No adverse effects noted
Upadacitinib	1	1	0	No adverse effects noted
Other specific treatments (acyclovir, clotrimazole)	3	3	0	No adverse effects noted

Certain findings were more frequently observed, including erythroderma, trichorrhexis invaginata, skin scaling, and atopic features [10-12]. Trichorrhexis invaginata was seen in 27 cases (79.41%). Atopic manifestations, such as asthma and allergies, were observed in 17 patients (50%). Desquamation was described in 16 cases (47.06%), alopecia in one case (2.94%), and ichthyosis linearis circumflexa (ILC) in 10 patients (29.14%). Erosions, hyperpigmentation, onychogryphosis, ectropion, vesicles, body odor, and vulvovaginal involvement were inconsistent findings whose manifestation was influenced by coexisting diseases [13,14].

Diagnostic methods included: genetic testing in 26 patients (76.47%), immunostaining of LEKT1 for six cases (17.65%), microscopy (hair or skin) in 12 cases (35.29%), and trichoscopy in four cases (11.76%) [15-18]. Access to advanced diagnostics varied from case to case. Initial misdiagnosis of NS occurred in four cases (11.76%).

Dupilumab was administered to eight patients (23.53%), secukinumab in six cases (17.65%), and pembrolizumab was used in one case (2.94%). Intravenous immunoglobulin (IVIG) therapy in nine patients (26.47%), omalizumab in two cases (5.88%), and JAK inhibitors (abrocitinib, tofacitinib, upadacitinib) in three cases (8.82%) with reported clinical improvement (reduced erythema) [19-30]. Supportive care involved topical corticosteroids, emollients, antihistamines, tacrolimus, and antibiotics (64.70%). Nutrition support for diet control and supplements was used for eight cases (23.52%). Acitretin was orally used for five cases (14.70%). Other specific treatments used in three cases (8.82%), such as acyclovir and clotrimazole, were seen in use due to associated diseases. Standard treatment is not yet established for this disease. Biological therapies, dupilumab and secukinumab, as well as IVIG, showed an association with symptom improvement across multiple cases of moderate to severe NS. However, none of the treatments explored achieved durable remission, and long-term efficacy data are limited. Use of emollients, corticosteroids, and antibiotics is important in symptom management, but does not provide treatment.

Clinical improvement was defined as reduced erythema, reduced itchiness, reduced scaling and dry skin, improved hair structure, and treatment of infections. Improvements were observed across various medication courses, but without a standardized treatment for NS, each case report consisted of a different regimen. Fourteen cases showed there were no signs of improvement (41.18%); eight cases (23.53%) showed relapse into the initial presentation, while six cases (17.64%) showed no reaction to treatments. There were cases that switched treatment due to failure to resolve symptoms, while others did not note treatment going forward. No patients achieved total curative remission, and no deaths were identified across the cases reviewed [31].

Discussion 

Genetic confirmation of biallelic variations of SPINK5 gene mutations remains the most reliable diagnostic tool; however, limited access restricts use [2]. In cases without genetic confirmation, the importance of recognizing distinguishing hallmark features of NS through detailed physical exams proved to be critical in order to avoid misdiagnosis with other dermatopathological diseases.

Alternative diagnostic methods included LEKTI and IL-36 immunostaining, which did aid in supporting diagnosis, but both were insufficient in providing a diagnosis individually [16]. LEKT1 technique demonstrated the protein’s deficiency in affected NS individuals, and IL-36 staining indicated that Th17-driven inflammation was occurring, but these techniques do not replace genetic confirmation, as they are more unreliable in definitive diagnosis. With other frequent findings, a similar situation is faced. Hair shaft abnormalities, particularly trichorrhexis invaginata, are a very valuable diagnostic finding, as the majority of cases showed this finding, but the absence of trichorrhexis invaginata does not exclude NS (atypical presentations exist). Scaling, desquamation, pruritus, and recurrent infections present due to the compromised skin barrier and immune dysfunction that characterize the disease, but they too are not reliable in making a diagnosis alone. Due to misdiagnosis of NS with other atopic dermatopathies, these findings work in congruence with one another in support of NS diagnosis; none of these findings alone is sufficient for diagnosis.

Clinical presentation of NS can vary reflecting the heterogeneity of dermatologic involvement within this disease; however, it was identified that erythroderma and eosinophilia presented in every patient reviewed. Elevated IgE is non-specific to NS, but does explain other symptoms that can occur, such as asthma, allergies, erythroderma, hair shaft defects, and atopic diathesis. Less common findings included ichthyosis linearis circumflexa (ILC) and skin manifestations, such as erosions, ectropion, and hyperpigmentation. Variability of presentation highlights the difficulty of diagnosis of NS [25].

Severe complications consisted of symptoms that progressed from dermatologic involvement. Underlying barrier dysfunction and immune dysregulation can lead to recurrent infections, electrolyte imbalances, and failure to thrive. Hypernatremia hospitalized an infant due to electrolyte imbalances, and such severe progression of NS could lead to a fatal outcome if not managed properly and early [19-21]. Recurrent infections along with a failure to thrive were reported, and while non-specific to NS, they can lead to mortality as well. One report described a squamous cell carcinoma (SCC) case, and while evidence is currently insufficient to state if there is an elevated malignancy risk, this occurrence shows a potential malignancy and morbidity risk. Therefore, oncologic vigilance is reasonable in long-term dermatologic treatment of NS, but the reviewed case reports do not provide sufficient data to establish protocols [30].

Current treatment of NS remains focused on symptomatic relief; however, the use of biologic agents described in this review shows promise, though data on long-term efficacy are limited. These findings emphasize the need for larger studies with standardized outcome reporting in order to establish treatment guidelines. Variability in NS presentation and treatment response highlights that early diagnosis and counseling is especially important in avoiding NS progressing to more severe forms that can be life-threatening. Individualized therapy and genetic counseling still seem to be the course of action for this disease.

Limitations of this study include a small sample size, variations in diagnostic techniques, heterogeneity of outcomes for patients, and failure to follow up/follow-up duration. Potential biases include detection, selection, and reporting. Fatal cases may be under-reported, while surviving cases may be disproportionately studied in higher detail. These constraints restrict the generalizability of findings, but do highlight the need for further studies and standardized reporting for improvement in NS treatment.

## Conclusions

Netherton's syndrome was shown to consistently present with erythroderma, ichthyosis, hair shaft abnormalities, and immune dysregulation to varying degrees. Complications of Netherton's, such as electrolyte imbalances, dehydration, or recurrent infections, will increase the severity of this disease, emphasizing the need for early identification and diagnosis. Molecular testing remains the most effective diagnostic modality, with a detailed physical exam remaining the mainstay diagnostic tool in low-resource areas. Symptomatic management with topical emollients, corticosteroids, and antibiotics has been successful with the currently proposed treatment therapies, which include biologics (dupilumab and secukinumab) and IVIG. However, these treatment options have varied in their lasting success.

Due to its rarity, NS remains shrouded in relative obscurity, and as such, early accurate diagnosis and consequently treatment remains a challenge. This systematic review used a 34-patient sample size to identify common NS presentations, successful diagnostic modalities, and successful therapies. The review proposes a pathway-dependent individualized therapy in union with genetic counseling as potentially the most reliable route to successful treatment. The review encourages future research endeavors with a larger sample size and a focus on lasting treatments.

## Appendices

**Table 5:** Summary of analyzed case reports for Netherton syndrome symptoms, treatments, and key findings.

Case report	Patient demographics	Dermatological manifestations	Treatment and outcomes	Key findings/conclusion
Author, year	Age, gender, ethnicity	Presentation, symptoms, lab tests, biopsy, imaging	Medications, surgery	Notable observations
Kovacheva et al. [1]	Six-year-old boy	The patient, diagnosed at birth with congenital ichthyosiform erythroderma, initially presented with generalized erythroderma and skin desquamation. By the fifth day of life, he developed neonatal infection and poor general health, which progressed to severe erythema, edema, and serous skin exudation involving the scalp and external auditory canals. The patient’s labs showed vitamin D deficiency, elevated IgE, and skin colonization with Proteus mirabilis. Biopsy revealed hyperkeratosis with parakeratosis and psoriasiform epidermal hyperplasia. Imaging showed delayed ossification of the wrist and palm, with bone age estimated at four years.	The patient initially received IV antibiotics, albumin, systemic antihistamines, and topical corticosteroids/emollients. After discharge, therapy included daily emollients, gentamycin for seven days, levocetirizine, cholecalciferol, and acitretin. Ongoing care consisted of regular emollient applications, sodium bicarbonate baths, and ocular treatment with tobramycin, dexpanthenol gel, and artificial tears.	Acitretin treatment initially provided slight improvement, but after three months the patient's condition worsened with severe skin thinning, erosion and persistent erythroderma, leading to discontinuation.
Moltrasio et al. [2]	Thirty-one-year-old woman	The patient's symptoms began in infancy with persistent eczematous eruptions and brittle, difficult-to-comb hair. By adulthood at age 31, she developed numerous erythematous, scaling lesions with annular and polycyclic patterns and an incomplete double edge of peeling scale, particularly on the arms, trunk, neck, and peri-areolar skin, often associated with itching and migratory recurrence. She also had multiple atopic comorbidities, including sensitization to dog and cat allergens, allergic rhinitis, conjunctivitis, food allergies (nuts and milk), and a history of asthma now in remission. Laboratory testing showed markedly elevated IgE, and skin biopsy demonstrated parakeratosis, microabscesses with eosinophils and neutrophils, dermal edema, and inflammatory infiltrates, with modest LEKTI staining on immunohistochemistry. Genetic analysis revealed a heterozygous frameshift mutation in SPINK5.	The patient had previously been treated with topical and oral corticosteroids as well as cyclosporine, which provided partial clinical improvement, but were followed by frequent relapses once discontinued.	Patient initially diagnosed atopic dermatitis. Netherton diagnosed based on genetic testing.
Wang et al. [3]	Thirty-five-year-old Chinese male	Initially developed erythematous skin lesions at three months of age, which gradually spread across his body and occasionally formed blisters. Dermatologic examination revealed diffuse erythema with desquamation and multiple flaccid vesicles, sparing the palms, soles, and oral mucosa. His scalp hair was yellowish, lusterless, and easily plucked, though trichorrhexis invaginata was absent on microscopy. Genetic analysis identified a novel homozygous frameshift mutation in SPINK5 in exon 26, not present in common databases or healthy controls, with the patient's parents and brother as heterozygous carriers. This C-terminal mutation likely explains the milder, atypical phenotype.	The patient was initially recommended treatment with oral acitretin, topical emollients, and topical corticosteroids according to European guidelines for congenital ichthyoses. Due to patient's concerns about acitretin’s side effects, he instead self-administered oral prednisone (10–15 mg/day) along with topical emollients and corticosteroid ointment for six months.	The case underscores that NS can present atypically, and absence of bamboo hair does not exclude diagnosis. SPINK5 mutation analysis is essential for confirming atypical cases and expanding the known mutational spectrum
Di Nora et al. [4]	One-month-old male	A male infant was evaluated for failure to thrive and feeding difficulties. He was delivered at 36 weeks after pregnancy with SARS-CoV-2 infection at the third trimester and gestational diabetes treated with alimental diet. After three days, he was admitted in the neonatal intensive care unit for dehydration and skin changes. Electrolyte analyses showed hypernatremia. He was discharged at two weeks of life, with normal value of natremia and indication of breastfeeding. Two weeks later, he was admitted to pediatric center. At the time of admission, presented with diffuse erythroderma. Skin coat with large areas of flaking in the region of the scalp - took on a crusty appearance with areas of alopecia. Muscle mass appeared hypotrophic and hypotonic. Presence of *Pseudomonas aeruginosa* on skin swabs	Treatment given for acute hypernatremia = careful IV fluid replacement to correct dehydration and gradually lower sodium levels (to avoid cerebral edema). Nutritional supplementation due to failure to thrive. Chronic management of NS involved supportive skin care (emollients, topical therapies)	Hypernatremia in Netherton syndrome infants is a serious and potentially life-threatening complication, resulting from skin barrier defects. Defects lead to excessive transepidermal water loss and vulnerability to infections. With patient’s feeding difficulties and increased metabolic demands, medical intervention was a necessity. Multidisciplinary management (pediatrics, dermatology, nutrition, nephrology) is needed for NS infant patients along with vigilant monitoring of hydration and electrolytes.
Merad et al. [5]	Seven-year-old female. Algerian (Sidi Bel Abbes, Algeria).	The patient presented from birth with recurrent erythroderma and desquamative skin lesions, later developing severe pruritus, scaling, eczematous patches, recurrent otitis, onychogryphosis, sparse brittle hair with loss of eyebrows/eyelashes, growth delay, and short stature. Examination showed erythematous scaly skin, brittle sparse hair, thickened nails, and ear canal debris. Hair microscopy revealed trichorrhexis invaginata, and biopsy showed non-bullous ichthyosiform erythroderma. Labs demonstrated mild leukocytosis, markedly elevated IgE, and *Candida parapsilosis* otomycosis.	Treatment included emollients, urea-based keratolytics, oral antihistamines, and topical corticosteroids for inflammation, along with clotrimazole ear drops for *Candida parapsilosis* otomycosis. Supportive care involved an allergen-free diet and vitamin D/calcium supplementation. Over 12 months of follow-up, the patient remained stable with no recurrence of pruritus or erythroderma, resolution of the ear infection, a 3 kg weight gain, and modest growth improvement, though long-term management is still required due to the chronic, incurable nature of Netherton syndrome.	In this case, otomycosis developed secondary to skin barrier defects and keratin debris accumulation in the ear canal, while growth delay and muscle weakness reflected systemic manifestations of Netherton syndrome. Consanguinity was identified as a risk factor, and the *Candida parapsilosis* infection highlighted the patient’s increased susceptibility to fungal infections due to impaired barrier function. Symptomatic management combined with targeted antifungal therapy led to clinical improvement; however, a long-term cure remains unattainable given the genetic basis of the disease.
Gordon et al. [7]	Male, South Asian (Indian) descent; patient that was followed from an age of four weeks, follow-up at 19 months, and again at five years of age	The patient's symptoms began shortly after birth. By day two of life, he exhibited generalized mild erythema, fine scaling, and peeling of the skin around his left foot, initially suspected to represent a staphylococcal infection. Over the following month, he developed a generalized erythroderma, with thicker adherent scaling notably on the scalp. Laboratory evaluation revealed marked eosinophilia. Total IgE levels were initially normal but increased significantly to >3000 kU/L by 19 months, accompanied by elevated allergen-specific IgE. Screening for other immune disorders, including Omenn syndrome and severe combined immunodeficiency, was negative, and plasma zinc and amino acid levels were normal. Skin biopsy at four weeks showed mild acanthosis, spongiosis, intraepidermal neutrophils with microabscesses, and a mixed dermal inflammatory infiltrate, consistent with subacute seborrheic dermatitis. Hair microscopy at five months revealed trichorrhexis invaginata. Immunofluorescence demonstrated normal LEKT1 expression. Genetic analysis identified a homozygous novel deletion of the entire coding region of the corneodesmosin gene (CDSN), confirming a diagnosis of generalized inflammatory peeling skin syndrome, with both parents being heterozygous carriers.	The infant initially received intravenous antibiotics for suspected staphylococcal infection associated with mild erythema, scaling, and peeling of the skin on day two of life. He was subsequently managed with regular emollients and topical 1% hydrocortisone. By 19 months of age, despite ongoing emollient therapy and trials of moderate-potency topical corticosteroids, there was no significant improvement in his skin condition.	Diagnosed with generalized inflammatory peeling skin syndrome (PSS), type B, caused by a novel homozygous deletion of the corneodesmosin gene (CDSN) spanning the entire coding region. NS was definitively excluded through immunofluorescence staining - which demonstrated normal LEKT1 expression - and targeted genetic testing, which showed only a heterozygous SPINK5 variant. This case emphasizes the diagnostic utility of targeted next-generation sequencing and genetic testing in identifying rare, genetically heterogeneous genodermatoses.
AlMoosawi et al. [8]	Two siblings affected: Patient 1: Fifty-eight-year-old Bahraini woman, Patient 2: Sixty-two-year-old male patient. Both born to consanguineous parents	Fifty-eight-year-old woman presented with generalized xerosis and ichthyosis on both of her lower legs extending to the dorsum of her feet. She also had extensive erythema with peeling over the neck, face, axilla, chest, thighs, and legs with islands of sparing. She complained of fragile hair, reporting that it has been short since childhood. She was diagnosed with psoriasis by a dermatologist in her past and given topical steroids. Skin biopsy was suggestive of pityriasis rubra pilaris (PRP). Sixty-two-year-old male presented with multiple peeling, erythematous scales, and plaques over his lower limbs and trunk. His condition was diagnosed as psoriasis by another dermatologist in the past, and he was started on topical treatment. Skin biopsy was suggestive of erythrokeratodermia variabilis. Both patients presented with generalized congenital erythroderma and scaling. Sparse, brittle hair. Trichorrhexis nodosa and pili torti were evident upon trichoscopy. Eye examination showed bilateral ectropion. Labs showed marked elevated IgE in both siblings. Genetic testing was performed, with the result showing the presence of gene mutation, SPINK5 (+) homozygous, classified as pathogenic mutation, confirming the diagnosis of NS.	Fifty-eight-year-old women with confirmed NS, received phototherapy + steroids, 62-year-old male received offered trial of Neotigason (acitretin, a systemic retinoid) or weekly phototherapy sessions as symptomatic treatment.	Consanguineous marriage increased risk of NS development. Both siblings were misdiagnosed (psoriasis or pityriasis rubra pilaris) and therefore treated with therapies aimed at those conditions and were ineffective for NS.
Hamza et al. [9]	Twenty-seven months, male child, born to consanguineous parents	Affected with generalised erythematous desquamative skin rash (erythroderma) from birth. At four months of age, the patient had normal IgG, IgA, and IgM levels. Repeated immune-panel testing indicated normal complement C3, C4 and total haemolytic complement levels. Normal lymphocyte count, mildly elevated eosinophil and normal IgE. The patient suffered recurrent infections from the age of one month, which increased in frequency and worsened in severity over time. Cultures from his blood, ears, cerebrospinal fluid, and eyes showed colonization by methicillin-resistant *Staphylococcus aureus*. Until the age of two years, he required peripheral red blood cell transfusion. At 27 months, presented with elevated IgE, and physical exam findings consisted of congenital erythroderma, brittle hair, growth issues. Hair shaft microscopy confirmed trichorrhexis invaginata. Skin biopsy consistent with ichthyosiform dermatitis. Genetic testing showed homozygous SPINK5 mutation.		Patient had an older male relative that had presented with similar symptoms and ultimately passed away. Relative developed sepsis in the NICU with coagulase-negative staphylococci and candida. The parents of the index patient revealed a history of infant deaths of the index patient’s paternal cousins, who had died of sepsis before the age of one and had received diagnoses of atopic dermatitis.
Moar et al. [10]	Two-year-old male child	The patient presented with chronic pruritic, erythematous, scaly lesions on hands, feet, knees, and flexural areas, along with fragile hair showing trichorrhexis invaginata. On follow-up, they developed fever and eczema herpeticum due to *Staphylococcus aureus* and HSV-1 infection, with concurrent SARS-CoV-2 RNA positivity. Additional findings included diffuse eczematous lesions, post-inflammatory hyperpigmentation, ichthyosis linearis circumflexa, elevated IgE, and genetic variants in FLG2 and SPINK5.	The patient was initially treated with topical tacrolimus, corticosteroids, and urea-based emollients without improvement. On readmission, systemic therapy with IV ceftriaxone and IV acyclovir, plus topical rifamycin, led to marked improvement in skin lesions. Despite SARS-CoV-2 positivity, no respiratory symptoms occurred.	First description of the SPINK5 c.882+1G>C mutation and its role in Netherton syndrome. Coexistence of SPINK5 and FLG2 mutations likely contributed to severe atopic manifestations. Highlights the value of trichoscopy for early diagnosis as well as genetic testing
Park et al. [11]	Two-month-male African-American	At approximately one month old, the patient presented with rash on the face. A month later (two-month-old child), at first clinical visit, severe eczema-like rash, periorbital swelling, bilateral extremity swelling, non-bullous ichthyosiform erythroderma, and failure to thrive. Following visit, moisturizing and amoxicillin suspension were given - rash worsened. Recurrent infections (skin cultures grew MRSA). Worsening rash, hair loss, poor feeding, hypoalbuminemia, increased IgE, transaminitis. Abnormal coagulation profile (PT, aPTT, INR all prolonged)	At one month of age: the pediatrician treated the patient's skin condition as eczema and encouraged the patient to continue aggressive moisturizing with over-the-counter lotions that included petroleum jelly and skin protectants that were approved for pediatric eczema. After one week, the patient returned to the outpatient clinic and did not show improvement. At two months, symptoms had worsened and patient was immediately hospitalized (first hospitalization). IV vancomycin and cefepime were administered. A nasogastric tube was also started to alleviate the patient's failure to thrive. Hypoalbuminemia, increased IgE, transaminitis. Pediatric dermatologist at the acute care facility changed the treatment to add topical triamcinolone, hydrocortisone, and ketoconazole. This treatment was deemed successful when the patient's skin began to clear	Initially difficult to distinguish between Netherton syndrome and severe eczema due to overlap (erythroderma, high IgE, recurrent infections, failure to thrive)
Abdalrheem et al. [12]	Two-year-old girl	Presented with generalized scaly skin lesions since birth. The lesions started from birth when the mother noticed scaling over her daughter’s scalp. A few months after birth, these lesions progressed to involve the face, neck, abdomen, and upper and lower limbs. Her two sisters had a similar problem since infancy, and her parents were first-degree cousins. A physical examination revealed that there were widespread serpiginous erythematous pruritic plaques, surrounded by a double-edged scales characteristic of ILC in the extremities. Eosinophilia with elevated IgE were found.	Cutaneous lesions were managed using topical medications such as moisturizers, antibiotic and corticosteroid.	Authors noted that scaly erythroderma that is unresponsive to the usual treatment of atopic dermatitis should raise a high index of suspicion for possible NS
Bertrand and Funaro [13]	Twenty-seven-year-old woman	The patient's initial symptoms, which began around age 13, included episodes of thick white vaginal discharge that was abundant at times, along with significant vulvar involvement from Netherton syndrome. Ichthyosis linearis circumflexa, trichorrhexis invaginata, and atopic diathesis with elevated IgE levels.	Systemic and topical antibacterials and antifungals, topical corticosteroids, pimecrolimus, crisaborole, emollients, zinc oxide paste, oral prednisone, intravenous and subcutaneous immunoglobulins (anti-TNF-alpha, anti-IL-17A), and dupilumab. Continuous oral contraceptives provided the most significant relief for both vulvovaginal discharge and generalized skin involvement.	Impaired skin and mucosal barrier explains her chronic vulvovaginal involvement, desquamation, and maceration in intertriginous areas. The patient developed vulvar lichen simplex chronicus, vestibulodynia, and hidradenitis suppurativa, highlighting the complex and multifaceted clinical spectrum of NS.
Kostova et al. [14]	Eight-year-old male	The patient presented shortly after birth with generalized skin erythema and fine scaling, noted at five days of age during admission to the neonatology ward. His skin condition was further complicated by a secondary infection, leading to an initial diagnosis of erythroderma ichthyosiformis congenita. Lab tests revealed a fivefold increase in liver transaminases, decreased serum albumin, creatinine, and uric acid levels, mild increases in D-dimers and fibrinogen, very low 25-OH vitamin D levels despite supplementation, elevated procalcitonin and C-reactive protein, and a markedly elevated serum IgE (>2500 IU/mL). Elevated IgE to multiple pollens, foods, and animal proteins, though the child had no clinical allergic symptoms or family history. Microbiological cultures isolated *Citrobacter* spp. from nasal and wound swabs and *Staphylococcus aureus* from throat and blood during hospitalization. Skin biopsy was not detailed, though NS diagnosis was confirmed genetically, typically supported by absent or reduced LEKTI expression.	High-dose IVIG therapy was later administered, leading to significant improvement in skin status, reduced infections, and growth gains, primarily through its immunomodulatory effects rather than IgG replacement	High-dose IVIG therapy produced substantial improvements of better skin status, fewer infections, normalized liver enzymes, decreased IgE levels, improved growth, and a reduced NASA score - without adverse effects. likely due to immunomodulatory mechanisms rather than IgG replacement. The case emphasizes IL-17A–blocking therapy could be considered for persistent inflammatory flares or relapses.
Temboonnark et al. [15]	Five-month Thai female; two-month Thai male	Five-month Thai female: Presented with exfoliative dermatitis. Shortly after birth developed congenital pneumonia with exfoliative dermatitis on face, trunk, extremities. Hx of severe infections (three cases septicemia, three cases pneumonia, one case acute b/l otitis media, infective endocarditis, and subdural empyema. Generalized erythroderma, weight and height below third percentile, skin is shiny, thick and scaly. Hair and eyebrows sparse, nail and mucosae normal; Two-month Thai male: Born at 34 weeks with generalized erythroderma and desquamation on first day, acute b/l otitis media at seven months. Recurrent ear infection 10 times in two years. Scaling erythroderma on face, trunk, and extremities. Hair and eyebrows sparse and fragile	Five-month Thai female: Treated with emollients and switched from cow milk to amino acid formula after IgE levels identified, but condition did not improve. After genetic testing, continued emollients and amino acid formula, and started oral retinoid; Two-month Thai male: Advised to avoid cow milk, soy, egg white, egg yolk. Treated with exfoliative moisturizers, acitretin, and hydrocortisone	Definitive diagnosis came from genetic testing showing SPINK5 mutation
Mintoff et al. [16]	Three-year-old male of Maltese-Caucasian	The patient’s initial symptoms began two weeks after birth with erythema and scaling of the skin, which progressively involved about 75% of his body surface area. He experienced recurrent staphylococcal cellulitis requiring oral and intravenous antibiotics, had hyper-IgEemia, and showed IgE-confirmed allergies to egg-white, soy, wheat, and nuts. Additionally, his hair was described as lusterless.	Patient’s ongoing symptoms were effectively managed with liberal use of bland emollients and as-needed antihistamines, which controlled his skin manifestations. For recurrent staphylococcal cellulitis, early skin cultures and targeted antibiotic therapy were used, successfully reducing both the frequency and severity of infection episodes.	Genetic analysis identified a novel homozygous intronic SPINK5 variant (c.2015+5G>A) disrupting the donor splice site of intron 21; segregation studies confirmed heterozygous carrier status in asymptomatic family members. Functional studies and computational analyses classified this variant as likely pathogenic, expanding the known SPINK5 mutational spectrum.
Pawlowski et al. [17]	Fifteen-year-old male; Twenty-four-year-old female	Fifteen year-old male: Presented long-standing rash, allergic comorbidities (allergic rhinitis, allergic conjunctivitis, food allergies), coin-shaped erythematous plaques with double collar of scale, trichorrhexis invaginata, and elevated IgE; Twenty-four-year-old female: Symptoms began in infancy with erythroderma - initially diagnosed as atopic dermatitis. Later developed pustular lesions with diagnosis revised to psoriasis. Presentation to office at 24-year-old with an established diagnosis of NS - serpiginous plaques with double-edged scale, trichorrhexis invaginata on eyebrow hairs. History of peanut allergy in female	Fifteen-year-old male: Prior treatment with methotrexate, cyclosporine, and dupilumab showed only transient improvement. Twenty-four-year-old female: IVIG, adalimumab, secukinumab, and dupilumab were tried. Best response was with IVIG	IL-36 expression was consistently high in both patients, supporting the role of Th17-driven inflammation in NS. IL-36 positivity suggests NS may respond better to treatments targeting Th17/IL-36 pathways (e.g., IL-17/IL-23 blockers), rather than solely Th2-targeted therapies like dupilumab. IL-36 immunostaining may help distinguish NS from eczema/atopic dermatitis but not from psoriasis
Mahajan et al. [18]	Seventeen-year-old woman	Patient exhibited long-standing severe atopic dermatitis (AD) that was refractory to standard treatments. Characterized by generalized erythema, scaling, and marked pruritus. She also had dry skin, generalized eczematous lesions, and ichthyosis linearis circumflexa (ILC). Hair abnormalities included short, sparse scalp hair with trichorrhexis nodosa observed on light microscopy. The patient had a past history of growth hormone deficiency and exogenous Cushing’s syndrome, and on examination presented with central obesity and prominent striae.	Treated with secukinumab, an anti-IL17A monoclonal antibody, administered at 150 mg subcutaneously once a week for five doses, followed by monthly maintenance. The patient showed significant clinical improvement. There was a notable reduction in pruritus, erythema, and scaling, with the ichthyosis severity score decreasing from 36 to 18 after four months of treatment. Her quality of life improved considerably, and no serious adverse events occurred. A transient appearance of verruca vulgaris was observed after four doses but resolved spontaneously within two months.	This case highlights the therapeutic potential of interleukin-17 inhibitor drugs like secukinumab in patients with NS.
Zhang et al. [19]	Chinese three-year-old boy	Generalized erythroderma from birth; never completely cleared. Developed into pruritic, erythematous lesions. The patient was initially misdiagnosed with severe atopic dermatitis (eczema) for two years, and topical corticosteroids temporarily helped these eruptions. The patient’s absolute eosinophil count and IgE were markedly elevated. Light microscopy of clipped scalp hair revealed the classic “bamboo hair” with a ball-and-socket appearance. Electron microscopy confirmed these findings, demonstrating bamboo-like nodules along the hair shaft. Sequencing (WES) of genomic DNA from peripheral blood identified candidate pathogenic variants in the SPINK5 gene. Sanger sequencing confirmed the c.80A>G mutation in both the proband and his father. In addition, quantitative real-time polymerase chain reaction (qRT-PCR) detected a large genomic deletion (chr5:147444834–147445034) in exon 2 of the SPINK5 gene, which was present in both the proband and his mother.	Patient was treated with intravenous immunoglobulin (IVIG) therapy for two months.	A reported case of NS caused by a large genomic deletion in the SPINK5 gene. The patient carried compound heterozygous mutations, and with sequencing, the spectrum of known SPINK5 mutations was expanded. The patient was initially misdiagnosed with severe atopic dermatitis for two years, drawing attention to the clinical overlap between NS and AD. Since the case demonstrated IVIG's effectiveness, this case provides valuable evidence for its role as a safe and effective treatment option in managing NS
Zelieskova et al. [20]	Two-year-old male	Patient presented with generalized erythroderma associated with skin desquamation. He gradually developed typical ichthyosis linearis circumflexa and suffered failure to thrive, recurrent respiratory tract infections, and hypotonia. During the first four months of life, he was hospitalized for severe hypernatremic, dehydration, colic, and failure to thrive. Elevated allergy markers, dysregulation in cellular immunity, and moderate hypogammaglobulinemia.	Intravenous immunoglobulin replacement therapy was administered monthly to treat the confirmed humoral immunodeficiency. Light microscopy of the hair revealed trichorrhexis invaginata. Molecular analysis using next-generation sequencing revealed a homozygous missense mutation in the SPINK5 gene: a previously unpublished and potentially pathogenic mutation c.1530CA (p.Cys510*), confirming the diagnosis of NS.	Patient of the report has an unreported homozygous mutation in the SPINK5 gene: variant c.1530CA (p.Cys510*). Expanded known mutations that can lead to NS.
Inaba et al. [21]	Twenty-six-year-old Japanese male	At birth, the patient had complications of red, scaling skin over entire body. Initially diagnosed as non-bullous congenital ichthyosiform erythroderma. Persistent rash, generalized erythema, scaly skin since infancy. In adulthood, rash worsened on skin with persistent pruritus despite treatments. Histopathology resembled psoriasis vulgaris.	Initial/ previous treatments consisted of moisturizers and antihistamines since birth with little to no significant improvement. Targeted therapy of Dupilumab with subcutaneous injection every two weeks. Dupilumab provided immediate and sustained relief of pruritus, which had been lifelong and previously resistant to antihistamines. This patient's response was sustained and complete, making it one of the stronger reported successes	Dupilumab success suggested IL-4 and IL-13 pathways play a role in Netherton syndrome pruritus, similar to atopic dermatitis. Histopathology resembled psoriasis vulgaris, suggesting overlap in cytokine pathways (IL-17/IL-36 involvement). Genetic confirmation of SPINK5 mutation was critical for diagnosis. Case highlights the overlap between NS and atopic dermatitis, suggesting dupilumab may fill an unmet need in Netherton syndrome treatment that typical atopic dermatitis treatments do not.
Vollmuth et al. [22]	Nine-week-old female infant	Patient presented with hypothermia despite external heat supply as well as hypernatremic dehydration. The entire skin was erythematous and scaly, biopsy showed subacute eczema. Molecular diagnostics using a primary genodermatosis gene panel revealed a known pathogenic homozygous SPINK5 splice site variant (NM_006846.4; c.1431-12G>A). In total, the diagnosis of NS was made.	The patient was administered dupilumab and within the first week of treatment, skin condition improved. Treatment continued with increasing doses. Hair started growing again. Shortly before the third dose, all supportive medication was discontinued. The daily nutritional intake could be increased. The patient started sleeping through the night without further signs of itching. Body weight increased from the third to the 10th percentile, body height increased from below the third percentile to the third percentile. No side effects occurred with a follow-up of 20 weeks.	This case showed the potential use of dupilumab in patients with NS, even those who are infants. Authors highlighted the importance of early diagnosis and immediate treatment for this pathology.
Johar et al. [23]	Saudi boy who was seven months old when presented symptoms-started treatment at age 18 months	Patient presented with generalized erythema at birth severe enough to require neonatal intensive care. Persistent erythema with scaling over the scalp and extremities, perioral fissuring, and pruritus throughout infancy. Hair abnormalities were also evident, with sparse, short, brittle scalp, eyebrow, and eyelash hair prone to breakage; trichoscopy confirmed the pathognomonic finding of trichorrhexis invaginata. The patient experienced frequent cutaneous infections requiring multiple hospital admissions.	The patient received a comprehensive therapeutic regimen consisting of intravenous immunoglobulin replacement therapy (IVIG), targeted food elimination, and dupilumab: IVIG was initiated at 18 months of age and was administered every four weeks due to recurrent cutaneous infections requiring antibiotics and hospital admissions. At six years of age, dupilumab was introduced as adjunct therapy to address persistent inflammation and pruritus, with the goal of improving skin barrier function prior to food reintroduction.	Success of treatments in causing remission of symptoms highlights the potential use of IVIG and dupilumab in Netherton treatment.
Blanchard et al. [24]	Sixteen-year-old male	Patient had skin issues since birth first starting with extremely dry skin that progressed overtime to erythematous plaques with a double-edged scale involving more than 90% body surface area at 17 months of age. At this time, he also had short, broken hairs, and trichoscopy revealed brittle hair with telescoping of the hair shaft. Based on the presence of both ichthyosis linearis circumflexa and trichorrhexis invaginata, the clinical diagnosis of Netherton syndrome was made. By the time he was aged five years, his disease was well controlled with only twice-daily emollient application and hydrocortisone 1% ointment, and he was lost to follow-up. At age of 16 years, patient presented for facial erythema and pain. Examination result was notable for erythematous scaly plaques distributed symmetrically on the nose, cheeks, nasolabial folds, and chin. Erythematous polycyclic scaly plaques were present on the abdomen and lower extremities. Diagnosis of Netherton syndrome was confirmed by exome sequencing, which showed heterozygosity for two pathogenic variants in the serine protease inhibitor Kazal type 5 gene. Biopsy of the facial rash showed focal parakeratosis, absent granular layer, and psoriasiform spongiotic epidermis.	Adalimumab, cyclosporine, narrow-band ultraviolet B, topical corticosteroids, pimecrolimus, econazole, itraconazole, ivermectin, acitretin, dapsone, doxycycline, prednisone, and omalizumab were all tried and all had a long-term inadequate response. Patient began receiving secukinumab once weekly, and at four-week follow-up he had remarkable improvement of both his facial and truncal rash. At his most recent follow-up, after almost three years of treatment with secukinumab, he had complete clearance of his facial erythema and only one mild flare of the polycyclic plaques on his trunk and extremities several months before	This report showed the benefit of secukinumab not just for the treatment of psoriasis as it is more commonly used for, but for NS as well. IL-17 immunoprofile in Netherton syndrome may provide a more specific therapeutic target in the treatment of this severe skin disorder.
Buchukuri et al. [25]	Twenty-year-old male	Initial symptoms began in infancy with generalized erythroderma, recurrent skin infections, chronic pruritus, and scaling. Patient also had asthma and multiple allergies. Trichoscopic examination revealed trichorrhexis invaginata, a highly specific finding. Laboratory workup demonstrated markedly elevated IgE levels and eosinophilia. Presentation as 20-year-old: Persistent diffuse erythema and scaling, severe pruritus, and recurrent bacterial skin infections. Trichorrhexis invaginata was confirmed. Genetic testing confirmed SPINK5 mutation.	Past therapies included topical corticosteroids, emollients, antihistamines, and various immunosuppressants. Therapy of report consisted of antibiotic treatment as the patient was started on low-dose acyclovir, amoxicil- lin-clavulanic acid, and methylprednisolone. Secukinumab was also administered - after two injections, the general condition of the patient improved, and after four injections, scaling and crusting was alleviated. Despite the overall symptom improvement, his trichorrhexis invaginata persisted during and after the treatment.	Secukinumab showed relief of symptoms in a patient with chronic NS. While symptoms did not resolve completely, this case supports the use of targeted biologic therapy.
Xiang et al. [26]	Four-month-old male child; nineteen-year-old woman	Four-month-old infant patient that even upon birth presented with entire body erythema, followed by the appearance of erythematous papules and scales after two days. Misdiagnosed with AD or psoriasis initially. Upon presentation at the time, patient had marked erythema on his head, face, trunk, and limbs, characterized by a circular or map-like pattern with thin scales and local scabs. Blood results indicated an elevated serum IgE level. The pathology of the skin lesion on the left upper arm revealed excessive keratinization, attenuation of the granular layer, mild psoriasis-like hyperplasia in the spinous layer, and little perivascular inflammation in the superficial dermis. Genetic testing was performed, which validated an SPINK5 mutation (two heterozygous mutations: c.2423C>T (p.T808I) and c.2468delA (p.K823Rfs*101)); nineteen-year-old woman went to the dermatology clinic with over 15 years of serrated erythema and serpiginous desquamation at the edges of a ring. The dermatological examination conducted at our hospital revealed severe hair breakage, sparse eyebrows, and underarm hair loss. Over the years, patient was repeatedly diagnosed with atopic dermatitis or psoriasis. Elevated eosinophils and IgE were noted.	Four-month-old male child: Following two months of dupilumab treatment, the skin flushing and ichthyic symptoms ameliorated, and the scoring atopic dermatitis index (SCORAD) decreased from 77.17 at baseline to 45.32 after two months of dupilumab treatment. The effect persisted until he reached three-year-old; nineteen-year-old woman: Received a combination treatment of secukinumab once weekly and dupilumab biweekly. After one month, the skin lesions exhibited substantial improvement. Combination therapy led to a SCORAD reduction from 73.2 to 48.1 over six months, while the Dermatology Life Quality Index (DLQI) improved from 13 to 9, reflecting the clinical enhancement observed. No extensive recurrence was noted throughout the six-month follow-up.	NS poses diagnostic challenges due to its variable presentation and overlapping features with common dermatoses like AD or psoriasis, often leading to prolonged misdiagnosis. These cases highlight the critical role of early recognition and genetic testing for definitive diagnosis and demonstrate that combination therapy with secukinumab and dupilumab effectively targets the mixed inflammatory pathways in NS, significantly improving both clinical symptoms and quality of life metrics.
Tang et al. [27]	Twenty-nine-year-old Chinese woman (Kunming, China)	This patient, with onset at birth of generalized erythroderma, developed recurrent red patches in childhood and progressive worsening over the past five years with extensive erythema, scaling, and erosions unresponsive to multiple therapies. On presentation, she exhibited diffuse serpiginous erythema, erosions with white scales, brittle “bamboo hair,” facial skin tightening with widened eyelid fissures severe pruritus. Laboratory evaluation showed marked eosinophilia and extremely elevated IgE. Dermoscopy confirmed trichorrhexis invaginata, histopathology revealed mild hyperkeratosis, acanthosis, spongiosis, and lymphocytic infiltration, and genetic testing identified compound heterozygous SPINK5 mutations.	Initial hospitalization with IV/topical glucocorticoids, oral antihistamines, topical antibiotics, and emollients produced only partial improvement. Subsequent outpatient therapy with omalizumab plus methylprednisolone yielded temporary improvement but relapse followed. Transition to abrocitinib achieved sustained clinical benefit, with progressive reductions in BSA involvement, EASI score, pruritus severity, IgE, and eosinophilia over six months, alongside denser hair growth though the bamboo hair phenotype persisted. Treatment was well tolerated without adverse effects, establishing abrocitinib as more effective than omalizumab in long-term disease control.	After eight months of therapy, the patient was lost to follow-up. Given the genetic basis of Netherton syndrome, there is currently no curative therapy, and management remains supportive and individualized. Emerging biologics such as Abrocitinib and Dupilumab have demonstrated efficacy in NS, while the role of Omalizumab remains insufficiently studied and requires further investigation in clinical settings.
Dodeja et al. [28]	Three-year-old boy	Patient had initial symptoms starting at birth and included waxing and waning generalized reddish-brown scaly plaques and recurrent infections. The skin lesions were described as multiple annular erythematous papules and plaques with double-edged scaling, with classic ichthyosis linearis circumflexa (ILC)-like lesions emerging around 18 months of age.	The patient initially received oral betamethasone, which failed to provide adequate symptom relief. Oral tofacitinib therapy was started alongside supportive care for eight weeks, resulting in a good therapeutic response at follow-up.	This case study highlights the successful management of Netherton Syndrome (NS) with oral tofacitinib. This case also describes the diagnostic challenges due to overlapping features with atopic dermatitis, and the potential of tofacitinib as a safer, effective therapeutic option. It also emphasizes the value of early hair examination for prompt diagnosis and encourages further research into tailored treatments for NS.
Sun and Chen [29]	Fourteen-year-old male	The patient's symptoms began shortly after birth and were characterized by xerosis, diffuse erythema, desquamation, pruritus, brittle and stunted hair, sparse eyebrows, irregular hair growth, notable body odor, and delayed development.	Initial therapy with antihistamines, emollients, and topical corticosteroids provided only partial and temporary relief of pruritus, with frequent relapses and persistent systemic involvement. Subsequent initiation of dupilumab led to early improvement in erythema and pruritus but failed to maintain long-term efficacy. Secukinumab was trialed next, producing transient clinical benefit that diminished over time, similarly to the dupilumab response. After other treatments, the patient transitioned to upadacitinib. This intervention produced early improvement, with reduction of body surface area involvement from 70% to <10% at eight weeks; however, disease control plateaued by the second month.	This case was the first to use Upadacitinib (JAK-STAT inhibitor) on a NS case. The benefits of Upadacitinib plateaued after two months, and therapy was discontinued due to limited sustained efficacy and increased body odor.
Cicek et al. [30]	Forty-day-old-male infant	Patient presented with recurrent sepsis, itchy eczematous dermatitis, scaling that had been present from birth. History of parents that were first cousins. Physical examination revealed xerosis and erythroderma with focal scaly plaques typical for ichthyosis linearis circumflexa. He had short, brittle and sparse scalp hair, eyelashes and eyebrows. Light microscopy of scalp hair demonstrated trichorrhexis invaginata. Weight, length, and head circumference were all below the third percentile. The eosinophil count, total serum IgE, and cow's milk protein-specific IgE were elevated. Serum immunoglobulin G (IgG) level was low. Histological findings showed subepidermal neutrophil-rich inflammation. A homozygous mutation in the serine protease inhibitor of the Kazal-type 5 (SPINK5) gene (c.410 +1G>A) was detected.	Despite emollients, antihistamines, antibiotics, topical corticosteroids, and intravenous immunoglobulin (IVIG), no improvement was found on the skin lesions. At six months of age, infliximab infusions were initiated on weeks zero, two, and six and continued every four weeks. Complete blood counts as well as kidney and liver function tests were obtained monthly without evidence of abnormality. Clinical improvement of both skin and scalp findings was appreciated. After a year of treatment, the skin and scalp rash had cleared and infliximab was discontinued. The patient continues to be managed with emollient therapy and has not had an exacerbation for the last 15 months.	This case highlighted the use of infliximab as its use was effective in improving the skin findings of our patient without causing adverse side effects. Infliximab may be a promising option for controlling NS.
Tos et al. [31]	Forty-four-year-old male	Diagnosed with Netherton syndrome in childhood. Presented with congenital ichthyosiform erythroderma, trichorrhexis invaginata, atopy. Presentation at age 44 to outpatient clinic with complaint of dry cough, dyspnea and squeezing chest pain of three-month duration. History of skin cancer, seasonal allergies, and elevated IgE. Severe skin inflammation with multiple nonhealing cutaneous lesions on face and scalp were present. Lesions were enlarging, ulcerated, and tender. Diagnosis of squamous cell carcinoma was made.	The patient used topical corticosteroid cream applied to his whole body daily after taking a shower to prevent the itching and reduce the redness since he was 13 years old. Patient also took antihistamines for seasonal allergies. He was started on Pembrolizumab as a treatment for his metastatic SCC. He had received 20 cycles of pembrolizumab, fixed three weeks apart. During his treatment he had not developed any immune related adverse events and showed clinical improvement after the third cycle. His cough and dyspnea were much better, and his appetite and weight improved during the treatment period. Immunotherapy course was well tolerated with no significant side effects in the patient and the PET scan and CT scan done at six and 12 months after starting pembrolizumab showed significant regression of his disease consistent with partial response according to the Response Evaluation Criteria In Solid Tumors (RECIST v. 1.1). Surgical excision of multiple SCC lesions was performed as well followed by reconstructive procedures to manage tissue loss after excisions. Patient continued forward with topical therapies and dressings for chronic skin disease.	This is a rare case of SCC in a patient with Netherton syndrome, which is not usually associated with high rates of skin cancer. Authors highlighted that chronic inflammation, skin barrier dysfunction, and long-term immunologic dysregulation in NS may predispose to malignancy. Authors stressed the importance of long-term monitoring for cancer in NS patients, especially those with persistent skin inflammation.
